# Type VI Collagen Regulates Endochondral Ossification in the Temporomandibular Joint

**DOI:** 10.1002/jbm4.10617

**Published:** 2022-03-10

**Authors:** Taishi Komori, Youngmi Ji, Hai Pham, Priyam Jani, Tina M. Kilts, Vardit Kram, Li Li, Marian F. Young

**Affiliations:** ^1^ Department of Health and Human Services Molecular Biology of Bones and Teeth Section, National Institute of Dental and Craniofacial Research, National Institutes of Health Bethesda MD USA

**Keywords:** BONE MATRIX, CHONDROCYTE AND CARTILAGE BIOLOGY, GENETIC ANIMAL MODELS, MOLECULAR PATHWAYS‐DEVELOPMENT, OSTEOARTHRITIS

## Abstract

For many years there has been a keen interest in developing regenerative treatment for temporomandibular joint–osteoarthritis (TMJ‐OA). Currently, there is no consensus treatment due to the limited self‐healing ability of articular cartilage and lack of understanding of the complex mechanisms regulating cartilage development in the TMJ. Endochondral ossification, the process of subchondral bone formation through chondrocyte differentiation, is critical for TMJ growth and development, and is tightly regulated by the composition of the extracellular matrix (ECM). Type VI collagen is a highly expressed ECM component in the TMJ cartilage, yet its specific functions are largely unknown. In this study, we investigated α2(VI)‐deficient (*Col6a2*‐knockout [KO]) mice, which are unable to secret or incorporate type VI collagen into their ECM. Compared with wild‐type (WT) mice, the TMJ condyles of *Col6a2*‐KO mice exhibit decreased bone volume/tissue volume (BV/TV) and a larger bone marrow space, suggesting the α2(VI)‐deficient condyles have a failure in endochondral ossification. Differentiating chondrocytes are the main source of bone cells during endochondral ossification. Our study shows there is an increased number of chondrocytes in the proliferative zone and decreased Col10‐expressing chondrocytes in *Col6a2*‐KO cartilage, all pointing to abnormal chondrocyte differentiation and maturation. In addition, RNA sequencing (RNAseq) analysis identified distinct gene expression profiles related to cell cycle and ECM organization that were altered in the mutant condyles. These data also suggest that bone morphogenetic protein 2 (BMP2) activity was deregulated during chondrocyte differentiation. Immunohistochemical analysis indicated an upregulation of Col2 and Acan expression in *Col6a*2‐KO cartilage. Moreover, the expression of pSmad1/5/8 and Runx2 was decreased in the *Col6a2*‐KO cartilage compared with WT controls. Taken together, our data indicate that type VI collagen expressed in the TMJ cartilage is important for endochondral ossification, possibly by modulating the ECM and altering/disrupting signaling pathways important for TMJ chondrocyte differentiation. Published 2022. This article is a U.S. Government work and is in the public domain in the USA. *JBMR Plus* published by Wiley Periodicals LLC on behalf of American Society for Bone and Mineral Research.

## Introduction

The temporomandibular joint (TMJ) is one of the most common tissues affected by osteoarthritis (OA), which can cause chronic pain and mandibular dysfunction ultimately affecting the quality of life. TMJ‐OA is manifested by degenerative changes in the TMJ condyle including erosion, flattening, subchondral cysts, and osteophyte formation^(^ ^1^, ^2^, ^3^, ^4^ ^)^; however, there is no effective regenerative treatment because the mechanisms regulating the TMJ development remain unclear.

Unlike the articular cartilage of the knee joint, the cartilage of the TMJ condyle is classified as a secondary cartilage and its articular surface is covered with fibrous tissue rather than hyaline tissue. The TMJ condyle is also unique because it develops through an endochondral ossification process that is different from that which occurs in the epiphyseal growth palate of the knee joint. Specifically, in long bones, the epiphyseal growth plate fuses after endochondral ossification process is complete, whereas in the TMJ the cartilage gets thinner but it is permanent and retains its function as articular cartilage over its lifetime.^(^ ^5^ ^)^

Endochondral ossification is a biological process which is tightly regulated by numerous growth factors and their signaling pathways. During endochondral ossification, chondrocytes in the TMJ undergo a multistep process of differentiation. Based on the location and proliferative and differentiative state, chondrocytes are classified into a superficial zone, proliferative zone, transitional zone, and hypertrophic zone, each characterized by their unique morphology and specific gene expression patterns. In vivo lineage tracing studies showed that chondrocytes in the TMJ cartilage can transdifferentiate into bone cells during subchondral bone formation and endochondral ossification.^(^ ^6^, ^7^, ^8^ ^)^ Thus, elucidating the mechanisms regulating chondrocyte differentiation is important to understand endochondral ossification, and it could provide new mechanistic insights to develop novel ways to regenerate the damaged condyle.

The TMJ cartilage contains an extracellular matrix (ECM) composed of collagen, noncollagenous proteins, and proteoglycans that all play an important role in facilitating interactions between cells and signaling molecules, and controlling chondrocyte differentiation during endochondral ossification.^(^ ^9^ ^)^ Type VI collagen is one of the major components of the ECM in the TMJ cartilage and is composed of three alpha chains [α1(VI), α2(VI), and α3(VI)].^(^ ^10^ ^)^ If any one of the alpha chains is deficient, the type VI collagen triplex is not secreted or incorporated into the ECM.^(^ ^11^, ^12^, ^13^ ^)^ Type VI collagen is both a structural and a signaling molecule, interacting with other matrix molecules to organize ECM structure and modulate biological processes. Although type VI collagen is highly expressed in the TMJ cartilage ECM, its exact function remains unclear.

The ECM regulates the spatiotemporal signals of numerous growth factors important to the chondrocyte differentiation process.^(^ ^14^, ^15^ ^)^ Specific factors including SRY‐box transcription factor 9 (SOX9), runt‐related transcription factor 2 (RUNX2), WNT, parathyroid hormone‐related peptide (PTHrP), and growth factors in the fibroblast growth factor (FGF) and transforming growth factor β (TGFβ) families, including bone morphogenetic protein (BMP) control chondrocyte proliferation and differentiation. The multifactorial process governed by all these components must be well coordinated to elicit normal endochondral ossification.

Previous studies examined the role of type VI collagen on cartilage using type VI collagen α1 chain–deficient mice and found there was a significant difference in skeletal development in knee and hip joints.^(^ ^16^ ^)^ Based on these findings we predicted it is possible that type VI collagen could have a role regulating skeletal development in other joints. In this study, we focused on the role of type VI collagen in endochondral ossification in the TMJ and found that there was a decreased bone volume/tissue volume (BV/TV) in the condyle of mice deficient in the α2 chain of type VI collagen (*Col6α2*‐knockout [KO]). This was accompanied by a large bone marrow space, arising from abnormal chondrocyte differentiation. Immunohistochemical analysis demonstrated abnormal Runx2 expression in the *Col6α2*‐KO chondrocytes leading to an altered activation of the Smad1/5/8 signaling pathway. Here we propose that loss of α2(VI) results in reduced endochondral ossification, caused by abnormal regulation of key signaling pathways that are important for chondrocyte differentiation.

## Materials and Methods

### Animal experiments

The *Col6a2*‐KO mouse strain used for this research was created from murine embryonic stem cells (ES cells) clone 12228C‐E10, generated by Regeneron Pharmaceuticals, Inc. (Tarrytown, NY, USA) in the KOMP Repository (https://www.komp.org/redirect.html) and the Mouse Biology Program (https://mbp.mousebiology.org/) at the University of California Davis and made strain of mice that were backcrossed to the C57B6J strain for five generations. Animals were housed under standard conditions (55% humidity, 12 hours day/night cycle, standard chow and free access to water) following the guidelines and approval of The National Institutes of Dental and Craniofacial Research Animal Care and Use Committee (protocol #18‐865). Male mice were used for analysis.

### Micro–computed tomography

For micro–computed tomography (μCT) analysis, mice were euthanized, mandibles from 1‐month‐old, 3‐month‐old, and 6‐month‐old wild‐type (WT) and *Col6α2*‐KO mice were dissected, fixed for 24 hours at room temperature in Z‐fix (Anatech, Ltd, Battle Creek, MI, USA) and then stored in 70% ethanol at 4°C. The three‐dimensional (3D) reconstructed images of mandibles were acquired using a μCT scanner (μCT 50; Scanco Medical AG, Bassersdorf, Switzerland) with the following parameters: 70‐kV X‐ray source voltage, 85 μA of intensity/beam current, power at 6 W, 300 ms integration time, with an image resolution of 6 μm. The 3D mandible images were differentially segmented by a global thresholding software, and BV/TV was measured using AnalyzePro software (AnalyzeDirect, Inc., Stilwell, KS, USA).

### Histology and immunohistochemistry

Dissected mandibles were fixed in Z‐fix for 24 hours, rinsed with PBS overnight and decalcified with 10% EDTA for 14 days. Samples were then washed and dehydrated through a graded ethanol and xylene series before paraffin embedding. Sections were cut at 5 μm, deparaffinized, stained with hematoxylin and eosin (H&E) and observed and scanned using an Aperio ScanScope scanner (Leica ICC50 W; Leica, Wetzlar, Germany).

Frozen sections of mandibles were prepared using Kawamoto's film method.^(^ ^17^ ^)^ Briefly, samples were embedded with Super Cryoembedding Medium (SECTION‐LAB Co. Ltd., Hiroshima, Japan) and cut to a thickness of 3 μm with a tungsten carbide blade after mounting the adhesive film onto the cut surface. Samples were fixed with 4% paraformaldehyde (PFA) for 10 minutes and stained with H&E. For immunohistochemical staining, specimens were fixed with 4% PFA for 10 minutes, followed by incubation with primary antibodies at 4°C overnight after blocking with 10% Normal donkey serum (Jackson ImmunoResearch Laboratories, Inc., West Grove, PA, USA) for 60 minutes at room temperature. The dilutions used are shown in Table 1. After washing, the specimens were incubated with secondary antibody Alexa Fluor 647 anti‐rabbit (Thermo Fisher Scientific, Waltham, MA, USA) or Alexa Fluor 488 anti‐mouse (Thermo Fisher Scientific) for 60 minutes at room temperature. All images were acquired using fluorescent microscopy with the same laser intensity, brightness, and contrast during image acquisition. Chondrocytes in proliferative zone of the TMJ cartilage were counted using ImageJ software (NIH, Bethesda, MD, USA; https://imagej.nih.gov/ij/) on H&E sections.

**Table 1:** The Use of Antibody Dilution

Name	Antibody	Company	Dilution
Type VI collagen	Anti‐Collagen Type VI	Fitzgerald	1:50
PCNA	AntioPCNA (D3H8P) Rabbit mAb #13110S	Cell Signaling	1:100
Col10	Anti‐Collagen X ab58632	Abcam	1:100
Acan	Anti‐Aggrecan AB1031	Millipore Sigma	1:100
Col2	Anti‐Collagen II MAB 8887	Millipore Sigma	1:100
Runx2	Anti‐RUNX2 (D1L7F) Rabbit mAb	Cell Signaling	1:400
pSmad1/5/8	Anti‐Phospho‐Smad1/5 (Ser463/465) (41D10) Rabbit mAb #9516	Cell Signaling	1:100

### RNA‐sequencing and analysis

Six‐week‐old WT and *Col6α2*‐KO condyles were dissected from right and left TMJs, immediately frozen in liquid nitrogen, and pooled at −80°C until 10 condyles from five mice were collected. They were then put into the center of a tissue tube (Covaris, Woburn, MA, USA) frozen in liquid nitrogen, and pulverized using the CP02 cryoPREP Automated Dry Pulverizer (Covaris). Total tissue RNA was extracted by using a TriPure (Sigma‐Aldrich, St. Louis, MO, USA)/RNeasy (QIAGEN, Hilden, Germany) hybrid extraction protocol. Sequencing libraries were prepared using a Nextera XT kit (Illumina, San Diego, CA, USA), individually barcoded, pooled to a 2nM final concentration, and sequenced on a NextSeq500 (Illumina) using 37 pair‐end (PE) reads. After sequencing, the base‐called demultiplexed (fastq) read qualities were determined using FastQC (v0.11.2) (http://www.bioinformatics.babraham.ac.uk/projects/fastqc/), aligned to the GENCODE M11 mouse genome (GRCm38.p4) and gene counts generated using STAR (v2.5.2a). Post‐alignment qualities were generated with QoRTS (v 1.1.6). An expression matrix of raw gene counts was generated using R (http://www.R-project.org) and filtered to remove low count genes (defined as those with <5 reads in at least one sample). The filtered expression matrix was used to generate a list of differentially expressed genes (DEGs) between the sample groups using three statistical methods: DESeq2, EdgeR, and Limma‐voom. DEGs (*Col6a2*‐KO versus WT, false discovery rate (FDR) adjusted *p* value <0.05) were considered for further analyses based on results from DESeq2. The top 700 DEGs by FDR‐adjusted *p* value rank were analyzed by Enrichr (https://maayanlab.cloud/Enrichr/) and upregulated DEGs (fold change [FC] >2 and FDR‐adjusted *p* value <0.05) and downregulated DEGs (FC < −2 and FDR‐adjusted *p* value <0.05) were subject to Ingenuity Pathway Analysis (IPA) (QIAGEN, Valencia, CA, USA) respectively.

### Statistical analysis

Statistical analyses were performed with unpaired Student's *t* test (Prism; V.9.0.0; GraphPad, San Diego, CA, USA). A statistically significant difference was considered as *p* value <0.05.

## Results

### Type VI collagen is highly expressed in the ECM of the TMJ cartilage

**Fig. 1:**
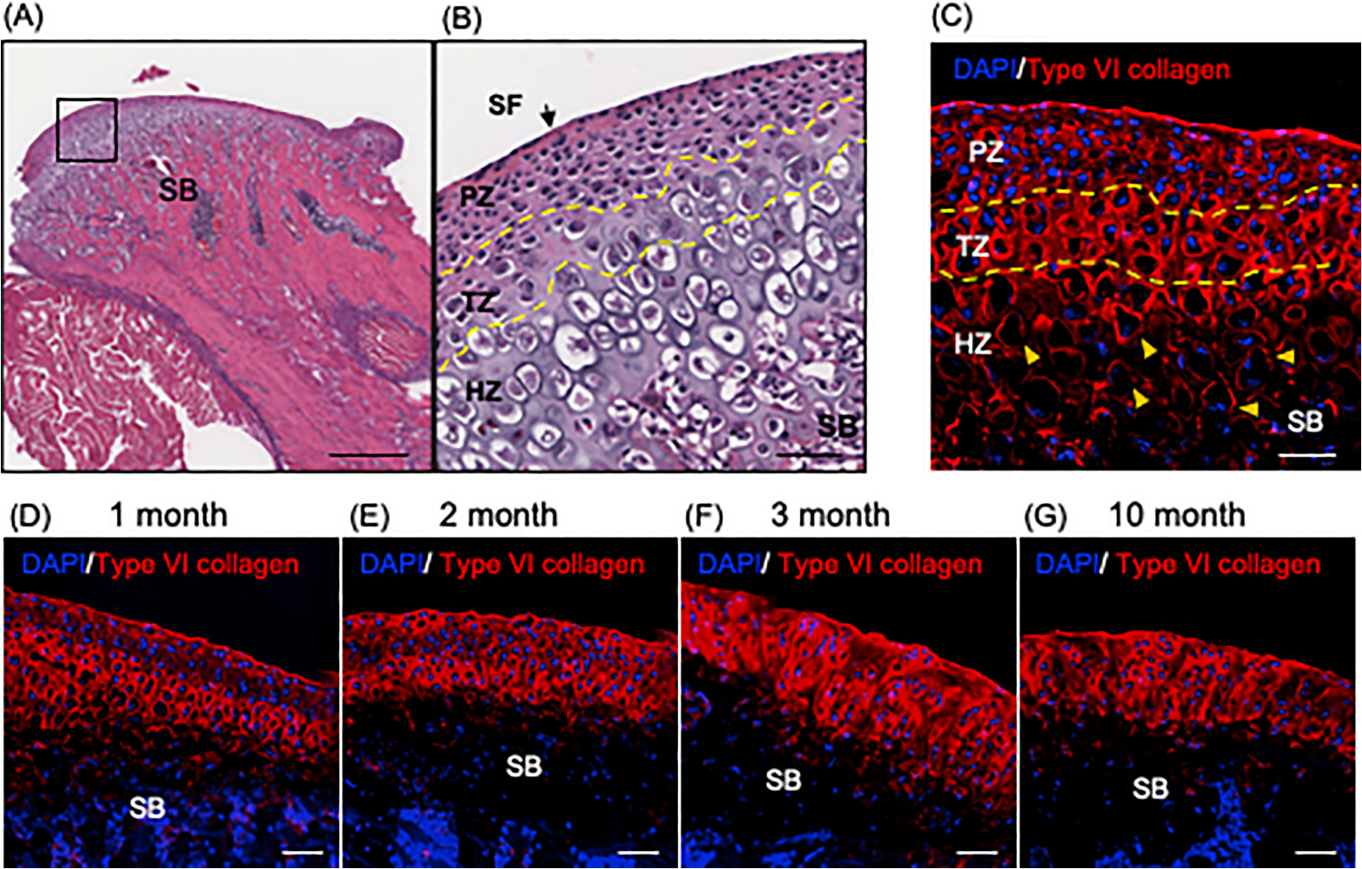
Type VI collagen is expressed in the TMJ cartilage ECM. Histological and immunohistochemical analysis of 1‐month‐old WT condyle. (*A*) H&E staining of the condyle. Magnified image of the boxed area is shown in *B*. (*C*) Immunofluorescent staining of type VI collagen in the TMJ cartilage. Arrowheads shows type VI collagen expression in the pericellular region. (*D*–*G*) Time series expression of type VI collagen in the TMJ cartilage: 1‐month‐old (*D*), 2‐month‐old (*E*), 3‐month‐old (*F*), and 10‐month‐old (*G*). Scale bars: (*A*) 400 μm, (*B*–*G*) 50 μm. Results are representative of at least three independent experiments. H&E = hematoxylin and eosin; HZ = hypertrophic zone; PZ = proliferative zone; SB = subchondral bone; SF = superficial zone; TZ = transitional zone.

To examine the expression of type VI collagen in the TMJ cartilage, we first defined the zonal cartilage organization from H&E staining of 1‐month‐old WT mice to delineate the superficial zone, proliferative zone, transitional zone, and hypertrophic zone (Fig. 1*A*,*B*). Sequential sections were used for immunohistochemical analysis of type VI collagen. The immunostaining revealed that type VI collagen was expressed in the ECM of the TMJ cartilage (Fig. 1*C*). Notably, the expression in the hypertrophic zone was restricted to the pericellular region (Fig. 1*C*, arrowheads). In mice, the TMJ condyle undergoes endochondral ossification, and continuously grows until 10 weeks of age thereupon it remains morphologically stable until 50 weeks of age. We performed time‐lined immunohistochemistry to examine type VI collagen expression during endochondral ossification and found that type VI collagen was expressed in the cartilage in 1‐month‐old and 2‐month‐old mice (endochondral ossification stage) (Fig. 1*D*,*E*) and continued to be expressed in 3‐month‐old and 10‐month‐old cartilage after the endochondral ossification process was completed. However, the discrete chondrocyte zonal organization is lost at these later stages (Fig. 1*F*,*G*).

### *Col6α2*‐KO TMJ condyles have reduced bone volume

**Fig. 2:**
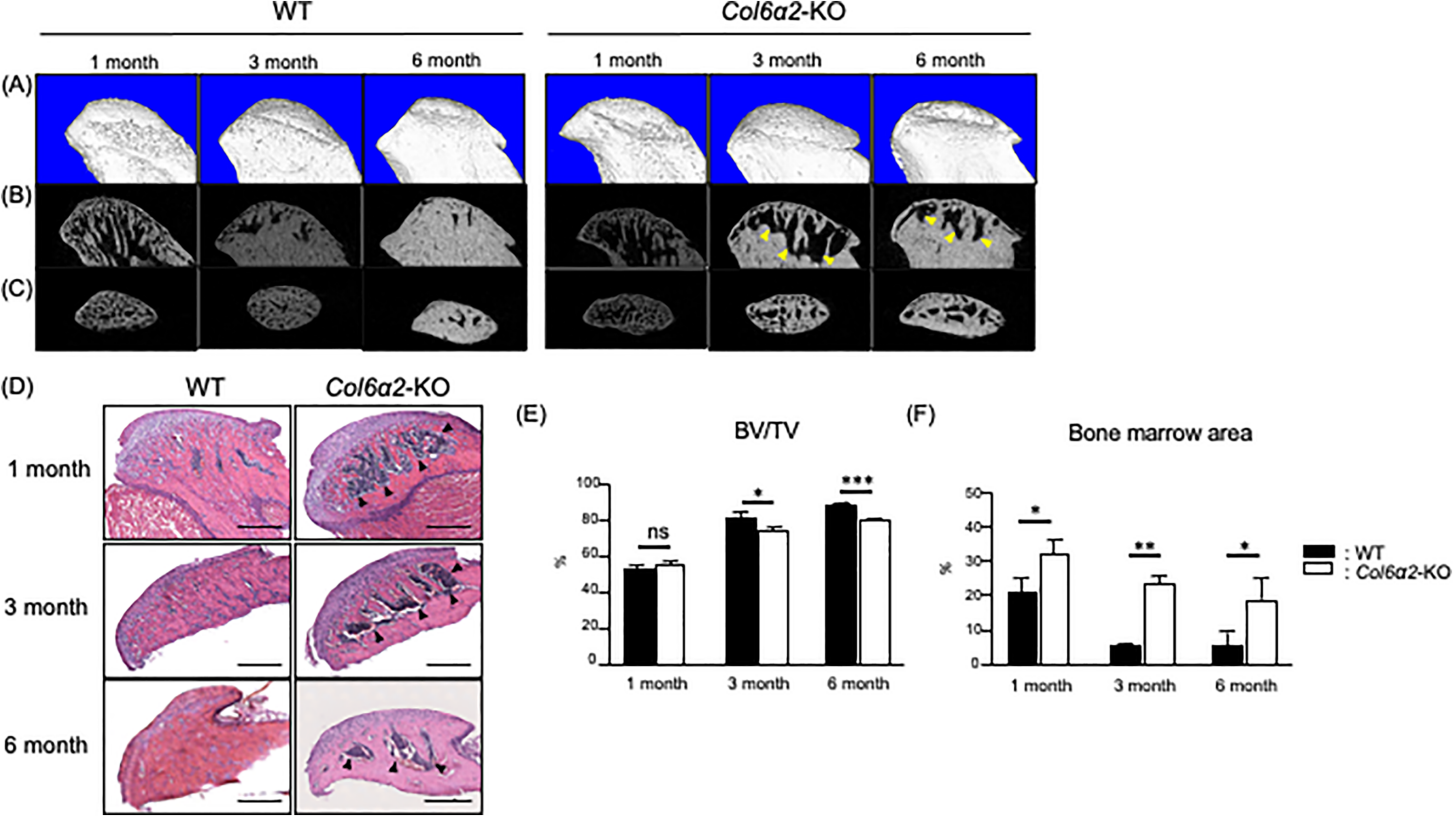
*Col6a2*‐KO condyle has reduced bone volume and larger bone marrow space. (*A*–*C*) μCT images from 1‐month‐old, 3‐month‐old, and 6‐month‐old WT and *Col6α2*‐KO condyles. (*A*) 3D reconstructed images of whole condyle, (*B*) mid‐sagittal slice, (*C*) axial slice. (*D*) H&E staining of 1‐month‐old, 3‐month‐old, and 6‐month‐old WT and *Col6α2*‐KO condyles. Arrowheads point to bone marrow spaces. Quantified data is shown in (*E*) bone volume fraction, and (*F*) bone marrow volume. Bars represent standard deviation. (*n* = 5). *p* value: *p < 0.05, **p < 0.01, ***p < 0.005, ns = not significant.

Because type VI collagen is highly expressed in the ECM of the TMJ cartilage, we used a α2(VI)‐deficient (*Col6α2*‐KO) mouse model that is unable to secrete any type VI collagen into its ECM^(^ ^18^ ^)^ to determine its function during endochondral ossification. μCT was used to visualize the mineralized tissues in WT and *Col6α2*‐KO condyles at 1 month, 3 months, and 6 months of age (Fig. 2*A*–*C*). Representative mid‐sagittal two‐dimensional (2D) slices show that the *Col6a2*‐KO condyles appeared to have larger marrow spaces in the subchondral bone (Fig. 2*B*, arrowhead). At 1 month of age, at the beginning of endochondral ossification, there were no significant differences in BV/TV; however, at 3 months and 6 months of age at after endochondral ossification, the BV/TV was significantly decreased in *Col6α2*‐KO TMJ condyles compared with WT condyles (Fig. 2*E*). As predicted from the μCT, H&E staining of histological sections of the condyles clearly showed that the *Col6α2*‐KO condyles had a large bone marrow space that replaced the subchondral bone compared with WT (Fig. 2*D*,*F*). These results suggested that α2(VI)‐deficient TMJ condyles have a failure in the progression of endochondral ossification.

### *Col6α2*‐KO TMJ condyles exhibited distinct gene expression profiles related to the cell cycle and ECM organization

**Fig. 3:**
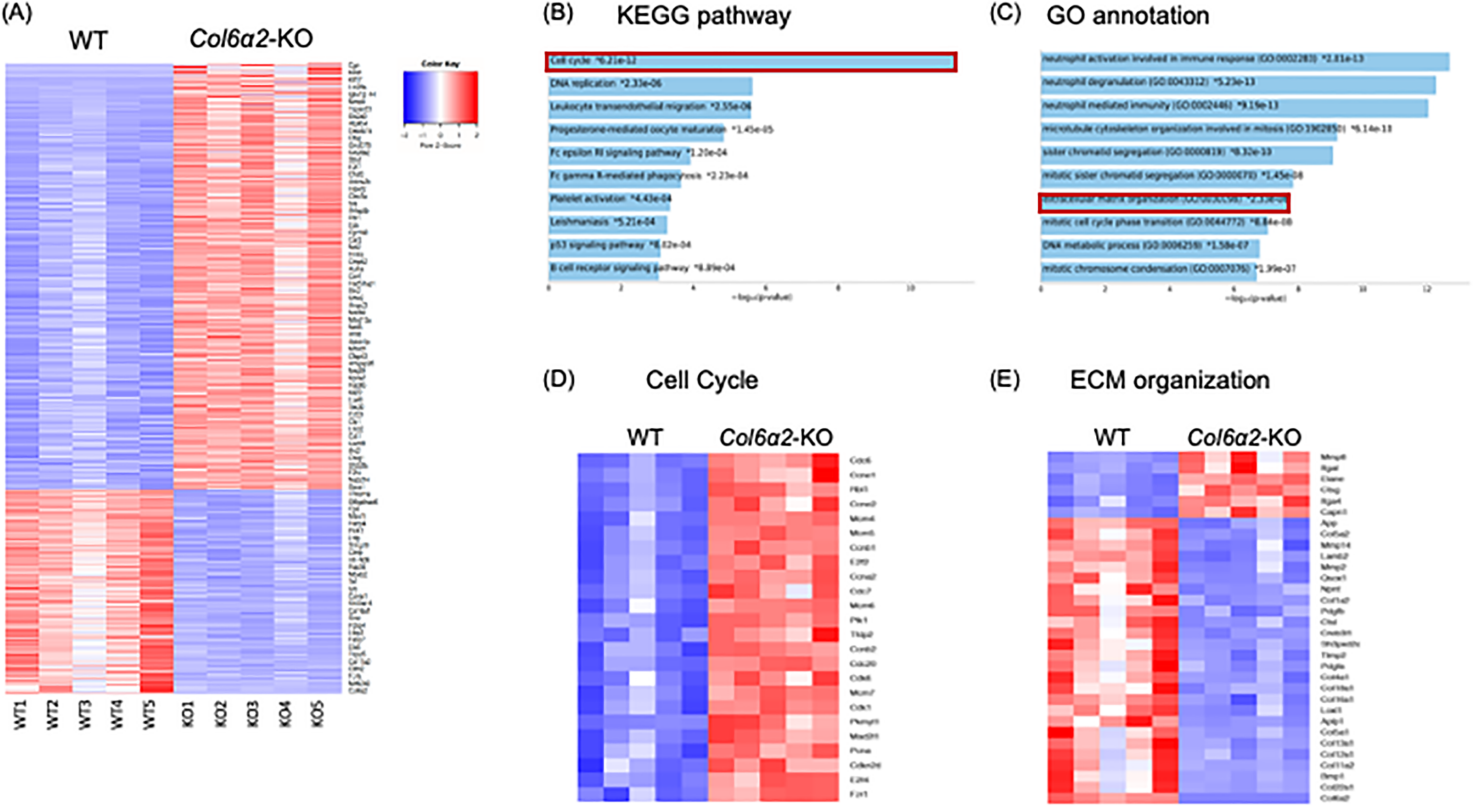
*Col6a2*‐KO condyle shows distinct gene expression profiles. Total RNA was extracted from 6‐week‐old WT and *Col6a2*‐KO condyles (*n* = 5). (*A*) Heatmap showing top 700 differentially expressed genes (DEGs: *Col6a2*‐KO versus WT, FDR‐adjusted *p* value <0.05). (*B*,*C*) Top 10 enrichment results from KEGG pathway analysis (*B*) and GO annotation analysis (*C*). (*D*) Heatmap showing genes associated with the cell cycle pathway, and (*E*) shows gene associated with extracellular matrix organization processes.

To try to elucidate the mechanistic basis of type VI collagen's role in TMJ cartilage, we performed RNAseq using total RNA extracted from 6‐week‐old WT and *Col6α2*‐KO condyles. Our RNAseq data revealed that 4975 genes were significantly differentially expressed between WT and *Col6α2*‐KO (FDR‐adjusted *p* value <0.05). Of these, 1343 genes were upregulated (FC > 2) and 744 genes were downregulated (FC < −2) in *Col6α2*‐KO compared with WT. Enrichr analysis showed the top ranked 700 differentially expressed genes (DEGs) (Fig. 3*A*) were associated with cell cycle (Fig. 3*B*) and extracellular matrix organization (GO:0030198) (Fig. 3*C*) as determined by the Kyoto Encyclopedia of Genes and Genomes (KEGG) pathway analysis and Gene Ontology (GO) annotation. The DEG list related to cell cycle and extracellular matrix organization is shown in Fig. 3*D*,*E*, respectively. Endochondral ossification in the TMJ is the formation of subchondral bone through chondrocyte differentiation, and the ECM plays an important role in facilitating interactions between cells and signaling molecules to regulate chondrocyte differentiation. Our RNAseq analysis showed that the α2(VI)‐deficient condyle had an increased levels of mRNA encoding genes related to cell cycle and significant changes in genes related to ECM organization.

### *Col6α2*‐KO TMJ chondrocytes showed abnormal differentiation

**Fig. 4:**
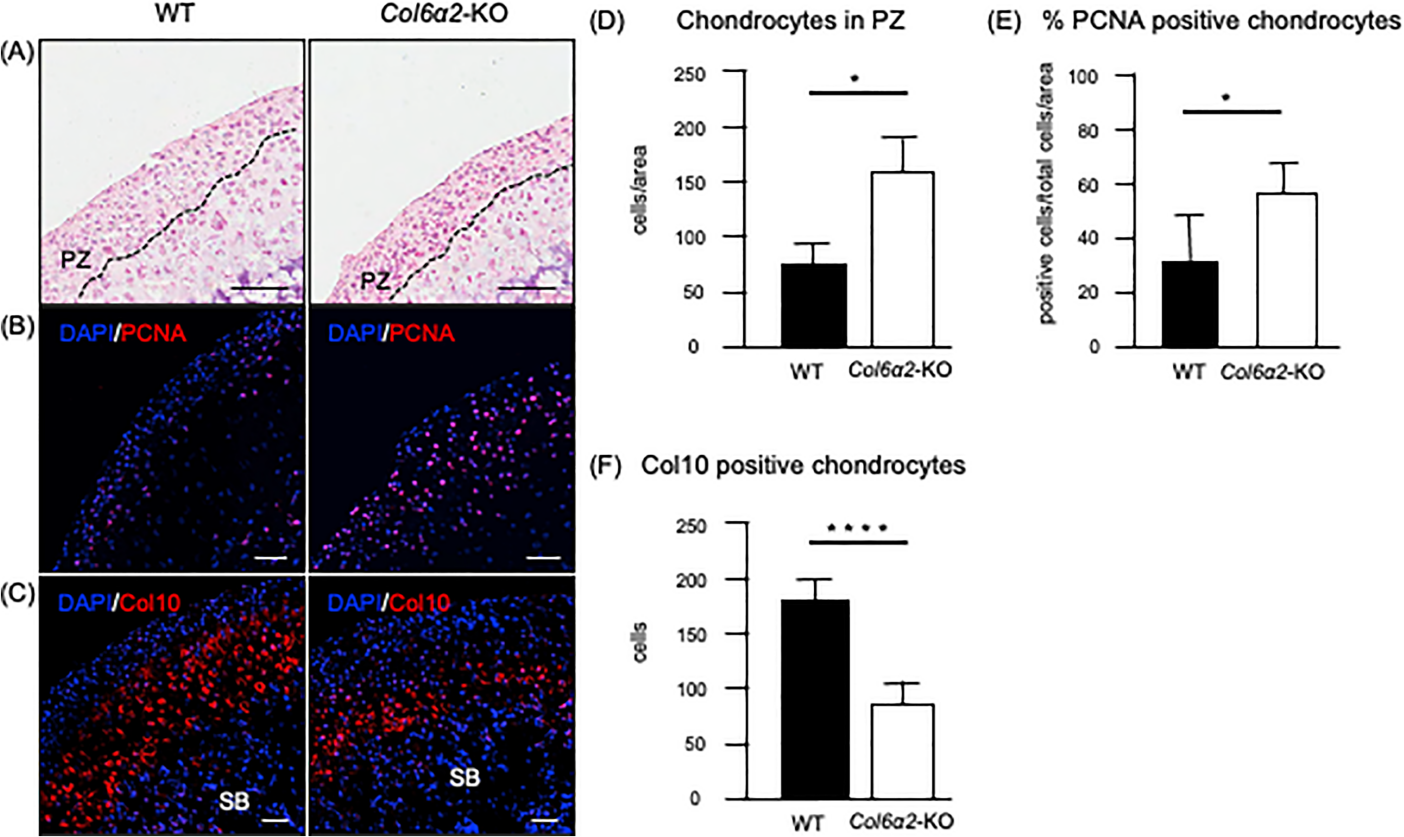
Type VI collagen deficient chondrocytes have increased proliferation and decreased type X collagen expression. (*A*) H&E staining of 1‐month‐old WT and *Col6α2*‐KO mice TMJ. (*B*,*C*) Immunofluorescent staining of the TMJ cartilage for PCNA (*B*), type X collagen (Col10) (*C*). Nuclei were stained with DAPI (blue). Scale bars: (*A*) 100 μm, (*B*,*C*) 50 μm. Results are representative of at least three independent experiments. (*D*,*E*) Quantification of the number of chondrocytes in the proliferative zone (*D*), PCNA‐positive chondrocytes (*E*) and Col10‐positive chondrocytes (*F*). Bars represent standard deviation (*n* = 5). *p* value: *p < 0.05, ****p < 0.001. PZ = proliferative zone; SB = subchondral bone.

Next, we focused on chondrocyte differentiation in the TMJ during endochondral ossification to specifically investigate the role of type VI collagen. At 1 month of age, the number of chondrocytes in the proliferative zone of *Col6α2*‐KO cartilage was increased compared with those found in WT cartilage (Fig. 4*A*,*D*). Immunohistochemical staining with proliferating cell nuclear antigen (PCNA) further confirmed this result, showing that the expression was significantly increased in *Col6α2*‐KO cartilage (Fig. 4*B*,*E*). This could explain the substantial changes in cell cycle gene expression in the *Col6a2*‐KO condyles revealed by DEGs analysis. When we stained sections with Safranin O we found that cartilage thickness was not changed. (Fig. S3A) To investigate mature chondrocytes located in the hypertrophic zone, immunohistochemistry for type X collagen (Col10) was performed. Our immunostaining revealed that the number of Col10‐expressing chondrocytes was decreased in *Col6α2*‐KO cartilage compared with WT controls (Fig. 4*C*,*F*). Together, these data imply there is abnormal chondrocyte differentiation in α2(VI)‐deficient cartilage during endochondral ossification.

### The expression of Col2 and Acan are affected in the *Col6α2*‐KO

**Fig. 5:**
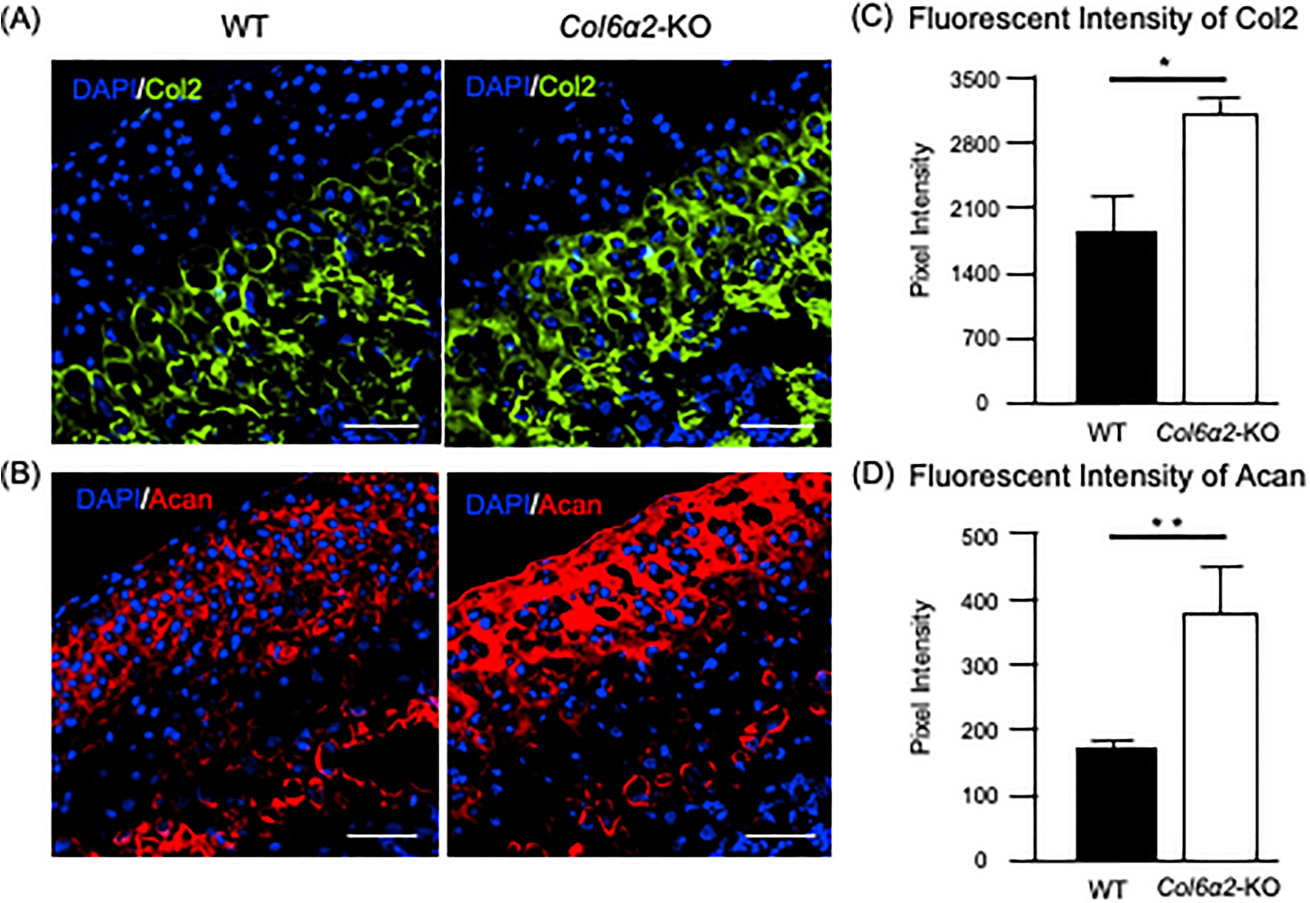
*Col6α2*‐KO TMJs have increased type ll collagen and Aggrecan expression. Immunofluorescent staining of the 1‐month‐old WT and *Col6α2*‐KO mice TMJ cartilage for (*A*) type II collagen (Col2) and (*B*) aggrecan (Acan). Scale bars = 50 μm. Results are representative of at least three independent experiments. Quantification of the fluorescent intensity of Col2 (*C*) and Acan (*D*). Bars represent standard deviation (*n* = 3). *p* value: *p < 0.05, **p < 0.01.

The ECM plays an important role in regulating chondrocyte differentiation by controlling signaling molecules. To examine the ECM components of the TMJ cartilage, immunohistochemistry was performed using type II collagen (Col2), a major structural fibrillar collagen and Aggrecan (Acan) the most abundant proteoglycan in the TMJ cartilage. The results showed both Col2 and Acan expression were upregulated in 1‐month‐old *Col6α2*‐KO cartilage compared with WT cartilage (Fig. 5*A*–*D*). These results suggest that type VI collagen has a role in organizing these ECM components in TMJ cartilage during endochondral ossification.

### The Smad1/5/8 signaling pathway is affected by lack of α2(VI) in the ECM

**Fig. 6:**
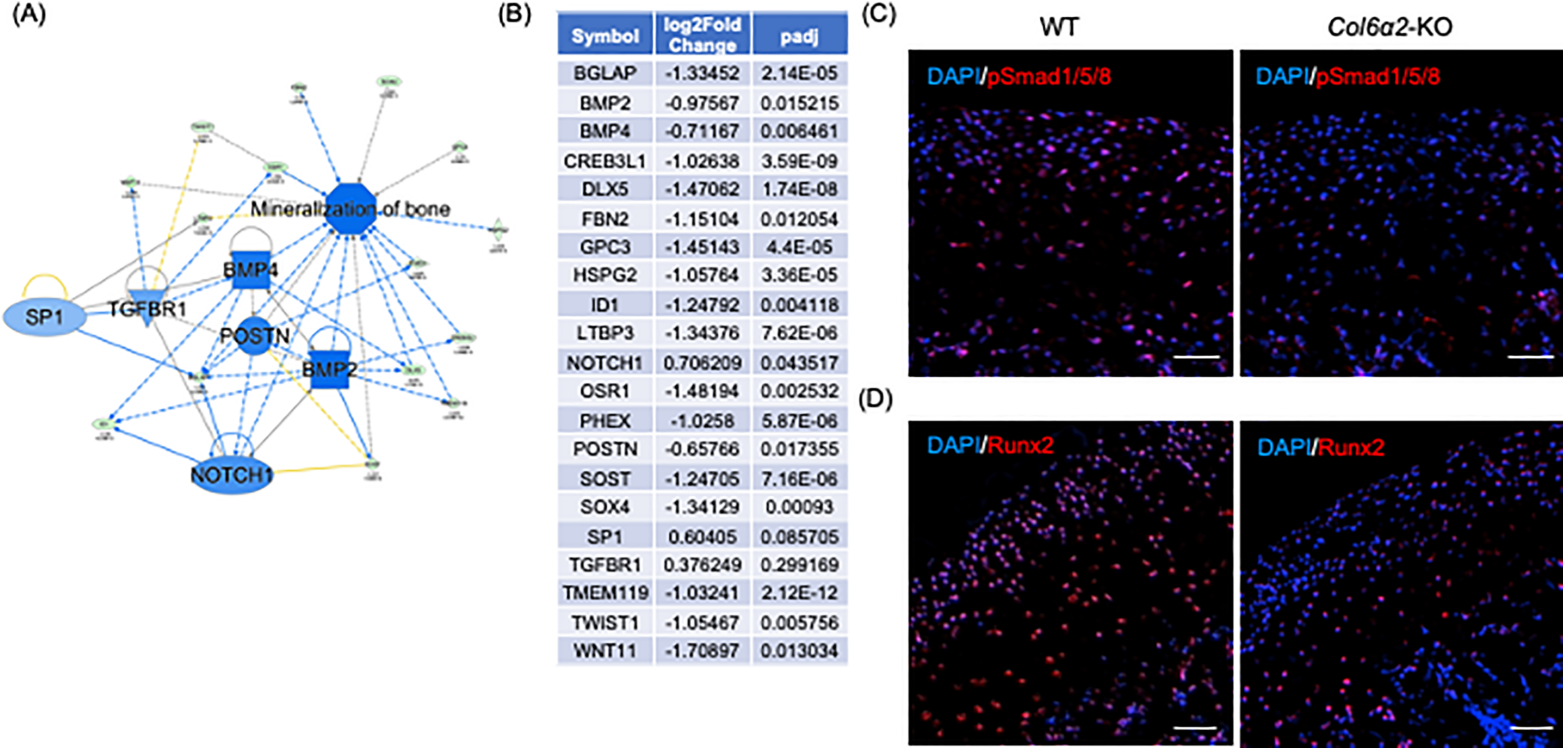
Type VI collagen deficiency affects bone mineralization via pSmad1/5/8 signaling. (*A*) Upstream regulatory network based on IPA analysis of downregulated genes. (*B*) List of genes associated with the biological function of “mineralization of bone.” Immunofluorescent staining for pSmadD1/5/8 (*C*) and Runx2 (*D*). Scale bars = 50 μm. Results are representative of at least three independent experiments.

To broaden our RNAseq analysis, we used the Ingenuity Pathway Analysis (IPA) with genes that were either upregulated (FC > 2, *p* value <0.05) or downregulated (FC < −2, *p* value <0.05) separately to identify possible upstream regulating genes and effected networks. The downregulated genes analysis uncovered several possible upstream regulators including Bmp2, Bmp4, Postn, Tgfβr1, Notch1, and Sp1, which are all known effectors of the downstream biological function “mineralization of bone” (Fig. 6*A*,*B*). Interestingly, from the IPA analysis of upregulated genes we also found Amphiregulin (Areg) as an upstream regulator gene and its regulated network includes MKi67, a gene associated with cell proliferation (Fig. S1A,B). Taken together, our bioinformatic analysis suggests that BMP2 signaling is affected by type VI collagen deficiency which could, in turn, affect subchondral bone formation during endochondral ossification. To confirm that downstream signaling of BMP2 is affected in the cartilage of *Col6a2*‐KO mice, we performed immunohistochemistry, and found that the expression of pSmad1/5/8 is decreased in *Col6a2*‐KO cartilage compared with WT control (Fig. 6*C*). In addition, we examined the expression of Runx2, a master gene that is governed, among other things, by the pSmad1/5/8 signaling pathway and regulates chondrocyte differentiation by suppressing chondrocyte proliferation and inducing maturation. Our staining of Runx2 expression was significantly decreased in *Col6a2*‐KO cartilage compared with WT control (Fig. 6*D*).

## Discussion

In the past several decades, many studies have been conducted in an effort to develop regenerative treatments for TMJ‐OA; however, there is still no consensual regenerative treatment because of the limited self‐healing ability of articular cartilage and the complex mechanisms regulating TMJ cartilage development. The ECM could be an important factor to consider in developing regenerative treatment because it enables cells to communicate through cell–cell and cell–matrix interactions^(^ ^7^ ^)^ and can also maintain homeostasis.^(^ ^14^ ^)^ Type VI collagen is a major ECM component in TMJ cartilage, yet its specific functions in the TMJ are largely unknown. In this study, we analyzed mice deficient in the α2 chain of type VI collagen (*Col6α2*‐KO), which are therefore unable to secrete the mature triple‐helical type VI collagen protein into their ECM.^(^ ^11^, ^12^, ^13^ ^)^ Our histological and μCT analyses show *Col6α2*‐KO condyles had a decreased BV/TV accompanied by larger bone marrow space, suggesting *Col6α2*‐KO condyles have a failure of endochondral ossification. In this study we show that type VI collagen has specific functions in chondrocyte differentiation by modulating ECM organization and controlling signaling pathways that are important for subchondral bone formation in the TMJ cartilage.

There are previous reports describing potential roles of type VI collagen on endochondral ossification in mineralized tissues other than the TMJ. When *Col6α1*‐KO mice were examined, delayed growth and ossification was observed at the femoral joint cartilage.^(^ ^6^ ^)^ A study by Christensen and colleagues^(^ ^16^ ^)^ showed *Col6α1*‐KO mice had significantly lower bone volume, trabecular bone volume, and trabecular thickness compared with WT mice. Furthermore, the increase in subchondral bone normally seen in WT mice with aging was not found in *Col6α1*‐KO mice, implying type VI collagen has a role of formation and maturation of bone.

The TMJ condyle is considered to be a secondary cartilage that undergoes endochondral ossification to form subchondral bone during its development. Our μCT analysis of the TMJ condyle aimed to evaluate bone formation through endochondral ossification showed a decreased BV/TV in the mutant TMJ condyles at 3 months and 6 months of age accompanied by a large bone marrow space. Interestingly, in aged mice (62–64 weeks old), there is still significant differences in BV/TV, suggesting there is impaired rather than delayed endochondral ossification in the mutant TMJ cartilage (Fig. S2). Although type VI collagen is expressed in the cartilage and appears to affect endochondral ossification, it is still unclear how it regulates bone growth and ossification. In vivo cell lineage tracing technology using cells expressing type II collagen (Col2)‐creER, aggrecan (Acan)‐creER, type X collagen (Col10)‐creER shows mature chondrocytes transdifferentiate into bone cells directly and not through the process of apoptosis as previously believed.^(^ ^8^, ^19^, ^20^ ^)^ These reports show that chondrocytes are the main source of bone cells for subchondral bone formation and suggests that understanding the mechanism of chondrocyte differentiation in this context could provide new insights for cartilage development and repair.

Chondrocyte differentiation is tightly regulated by the ECM.^(^ ^14^, ^21^, ^22^ ^)^ Col2 is the major structural fibrillar collagen of the cartilage that provides a highly ordered meshwork scaffold for chondrocytes and other ECM molecules. Mutations in the *Col2α1* gene have been characterized in several chondrodysplasias, including achondrogenesis (OMIM #200610) and spondyloepiphyseal dysplasia (OMIM #183900),^(^ ^23^, ^24^ ^)^ that vary in severity from perinatally lethal to mild disease. From these reports, it is clear that Col2 has a critical role in organizing cartilage by enhancing chondrocyte differentiation and affecting cell shape.^(^ ^25^ ^)^ Aggrecan (Acan) is a major cartilage proteoglycan that provides a scaffold to modulate proper interactions between cells and signaling molecules. Acan deficiency in mice leads to severe dwarfism and premature death, pointing to its essential role in skeletal development.^(^ ^26^ ^)^ Chondrocytes deficient in Acan display abnormal arrangement, reduced proliferation rate, and prominent cell death. These abnormalities come from altered Indian hedgehog (Ihh), FGF, and BMP signal transduction.^(^ ^14^ ^)^ Our immunostaining showed both Col2 and Acan expression were upregulated in *Col6a2*‐KO cartilage compared with WT cartilage. Our data also showed the number of chondrocytes in the proliferative zone was increased, whereas mature Col10‐expressing chondrocytes were decreased. These results, suggest that lack of α2(VI) affects chondrocyte differentiation via the alteration of major cartilage ECM components.

Bmp2 is a member of Tgfβ superfamily that plays essential roles in many developmental processes including osteoblast and chondrocyte differentiation.^(^ ^27^, ^28^, ^29^, ^30^ ^)^ As an osteochondrogenic factor, Bmp2 activates Bmp type 1 receptors and Smad1/5/8 signaling pathways to promote chondrocyte differentiation and endochondral ossification.^(^ ^31^, ^32^, ^33^, ^34^ ^)^ In the cartilage, Bmp2 is involved in different stages of chondrogenic differentiation. During early stages of chondrocyte differentiation, it promotes differentiation by regulating the expression of Sox9, whereas at later stages it induces proliferation and inhibits chondrocyte maturation.^(^ ^35^, ^36^, ^37^, ^38^ ^)^ Bmp is also involved in multiple stages of endochondral ossification by regulating Runx2 expression. Runx2 is an essential factor for skeletal development and is expressed in osteoblast progenitors.^(^ ^39^ ^)^ It is also expressed in chondrocytes^(^ ^40^ ^)^ and has been shown to be involved in multiple stages of cartilage development.^(^ ^41^ ^)^ Studies indicate that Runx2 regulates the expression of type X collagen in hypertrophic chondrocytes,^(^ ^42^ ^)^ and that maturation of chondrocytes is delayed in Runx2 KO mice.^(^ ^43^, ^44^ ^)^ These findings suggest that Runx2 positively regulates chondrocyte maturation and endochondral ossification. Taken together, Bmp2 and Runx2 have essential roles in endochondral ossification by regulating chondrocyte differentiation, and these key factors are controlled by multiple signaling cascades. Previous studies on cartilage development and our RNAseq data guided us to focus on Bmp2 signaling as potential mechanism leading to the reduced endochondral ossification seen in the absence of type VI collagen. IPA analysis identified Bmp2 and Bmp4 as upstream factors in our RNAseq data set, both known as important players in mineralization of bone. In addition, we found that in α2(VI)‐deficient cartilage, pSmad1/5/8 expression, a downstream effector of BMP2, was decreased, as was Runx2 expression, which is downstream of pSmad1/5/8 signaling pathway. Therefore, one molecular basis for the failure of the mutant condyles to differentiate into bone could be that the lack of α2(VI) affects Bmp2s ability to control subsequent downstream pathways affecting Smad1/5/8 signaling and its effectors.

Previous work showed type VI collagen may have mechanical and cytoprotective roles in the regulation of TMJ‐OA. A study by Alexopoulos and colleagues^(^ ^6^ ^)^ showed that *Col6α1*‐KO mice had accelerated joint degeneration in the femoral head leading to early development of OA as well as delayed ossification. Yotsuya and colleagues^(^ ^45^ ^)^ showed changes in the distribution of type VI collagen and Neuron/Glial Antigen 2 (NG2), a cell membrane proteoglycan that binds to type VI collagen, in the TMJ during surgically induced osteoarthritis. The authors suggest that type VI collagen could be mediating cartilage degeneration through interactions with NG2. Interestingly, using chondrocytes isolated from the TMJ, Chu and colleagues^(^ ^46^ ^)^ showed that pretreatment with type VI collagen reduced the interleukin 1β (IL‐1β) induced upregulation of genes related to matrix degradation, including matrix metalloproteinase 3 (MMP3), MMP9, and MMP13, and inhibited downregulation of the critical ECM component Acan. This implies that type VI collagen has chondroprotective mechanism against inflammation. Concerning our results showing there is increased PCNA expression in *Col6α2*‐KO cartilage, it is also interesting to note that Pfander and colleagues^(^ ^47^ ^)^ found PCNA expression was activated in osteoarthritic (OA) cartilage in humans suggesting that the absence of type VI collagen may be linked to the pathology of OA. In the present study a comprehensive analysis of OA in the TMJ of *Col6α2*‐KO judged by bone parameters from μCT or from Mankin score evaluation was not carried out. It is possible that type VI collagen modulates TMJ‐OA during aging or in TMJ‐OA induced by trauma or surgery; however, further experiments will be needed to clarify this point.

Type VI collagen binds to a wide range of ECM molecules, including type IV collagen,^(^ ^48^ ^)^ decorin,^(^ ^49^ ^)^ biglycan,^(^ ^50^ ^)^ perlecan,^(^ ^51^ ^)^ and NG2 proteoglycan,^(^ ^52^ ^)^ and it also associates with other collagens, potentially through interactions with proteoglycans.^(^ ^53^, ^54^ ^)^ In addition, our previous study in long bones showed type VI collagen binds to Tnfα and works by harnessing its activity.^(^ ^18^ ^)^ It is possible that type VI collagen directly binds to Bmp2 in the cartilage; however, additional biochemical experiments would need to be carried out to prove this point. Regarding the integrity of the ECM, we show here distinct changes in gene expression profiles related to ECM organization. The genes affected include other collagens (eg, *Col1α2*, *Col4α1*, *Col11α2*, *Col12α2*, *Col13α2*, *Col18α1*) as well as genes that affect collagen (including *Timp2*, *Mmp2*, and *Mmp8*). Taken together we conclude that type VI collagen potentially works as a networking factor that modulates cartilage ECM molecules that subsequently control signaling pathways important for chondrocyte differentiation.

In conclusion, we show for the first time that type VI collagen expressed in the TMJ cartilage has an important role in the formation of subchondral bone during endochondral ossification by regulating chondrocyte differentiation. In addition, we show type VI collagen may work as a networking factor, modulating cartilage ECM molecules that subsequently control the pSmad1/5/8 signal transduction pathway and Runx2 expression both known to be important for chondrocyte differentiation. Further studies are necessary to elucidate the molecular link between type VI collagen and signaling molecules needed for chondrocyte differentiation and, further, to determine how the progression from immature chondrocytes to mature chondrocytes and ultimately bone cells is affected in α2(VI)‐deficient TMJ cartilage. Another important next step to this study will be to evaluate the expression of type VI collagen during early stages of TMJ development to determine what role it plays in earlier stages of TMJ formation.

## Authors contributions

**Taishi Komori:** Conceptualization; data curation; formal analysis; investigation; methodology; writing – original draft. **Youngmi Ji:** Investigation. **Hai Thanh Pham:** Investigation. **Priyam Jani:** Investigation; methodology. **Tina M. Kilts:** Resources. **Vardit Kram:** Investigation; writing – review and editing. **Li Li:** Investigation. **Marian F. Young:** Conceptualization; funding acquisition; project administration; resources; supervision; writing – review and editing.

## Conflict of Interest

The authors declare no competing financial interests.

### PEER REVIEW

The peer review history for this article is available at https://publons.com/publon/10.1002/jbm4.10617.

## Supporting information


**Fig. S1** (A) Upstream regulatory network generated by IPA using upregulated genes (FC > 2, *p* value <0.05) showing AREG network associated with cell proliferation. (B) Heat map of genes related to AREG effect.

**Fig. S2** (A) Representative 3D rendered images and mid‐sagittal sections of aged condyles (61 to 64 week old mice). (B) Quantified data for BV/TV. Bars represent standard deviation (5 WT and 4 *Col6a2*‐KO). *p* value: **p < 0.01.

**Fig. S3** (A) Safranin O staining in the cartilage of WT and *Col6a2*‐KO. ns: not significant.
