# Supplementary figures and images for: Type VI Collagen Regulates Endochondral Ossification in the Temporomandibular Joint
Source: JBMR Plus. 2022 Mar 10;6(5):e10617. doi: 10.1002/jbm4.10617 (PMC9059467; doi:10.1002/jbm4.10617)

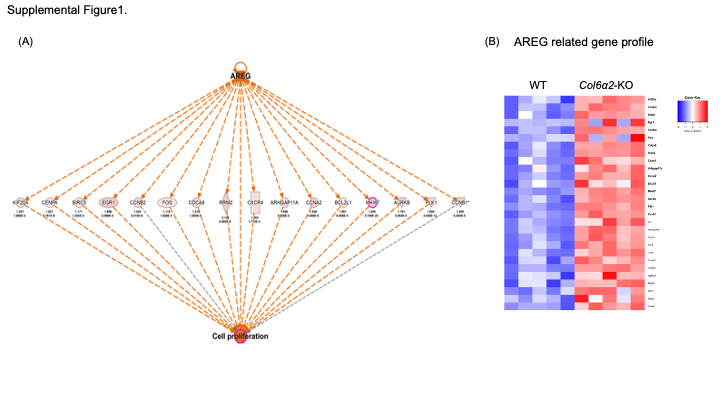

Supplement: Supplementary file 1 — Fig. S1 (A) Upstream regulatory network generated by IPA using upregulated genes (FC > 2, p value <0.05) showing AREG network associated with cell proliferation. (B) Heat map of genes related to AREG effect. [file JBM4-6-e10617-s003.tiff]

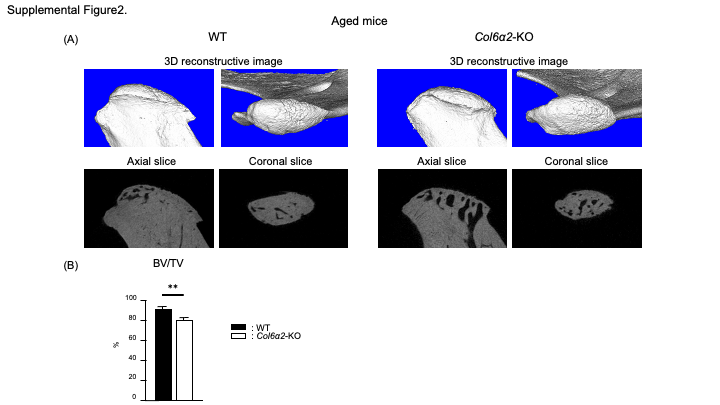

Supplement: Supplementary file 2 — Fig. S2 (A) Representative 3D rendered images and mid‐sagittal sections of aged condyles (61 to 64 week old mice). (B) Quantified data for BV/TV. Bars represent standard deviation (5 WT and 4 Col6a2‐KO). p value: **p < 0.01. [file JBM4-6-e10617-s002.tiff]

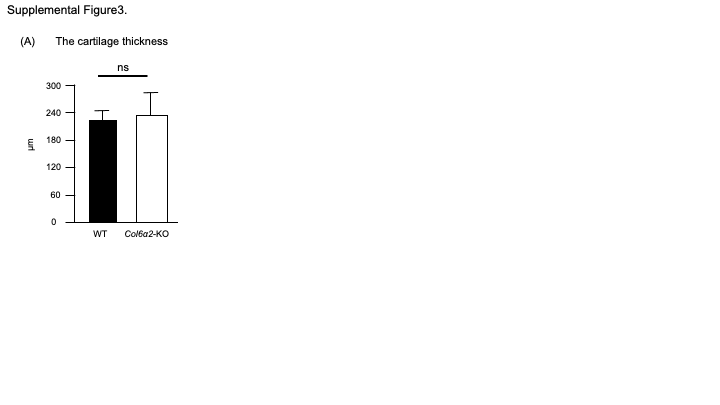

Supplement: Supplementary file 3 — Fig. S3 (A) Safranin O staining in the cartilage of WT and Col6a2‐KO. ns: not significant. [file JBM4-6-e10617-s001.tiff]
